# Construct-Specific and Timing-Specific Aspects of the Home Environment for Children’s School Readiness

**DOI:** 10.3389/fpsyg.2020.01959

**Published:** 2020-08-05

**Authors:** Yemimah A. King, Robert J. Duncan, German Posada, David J. Purpura

**Affiliations:** Department of Human Development & Family Studies, Purdue University, West Lafayette, IN, United States

**Keywords:** home learning environment, school readiness, early childhood education, language ability, math skills, externalizing behaviors, parent-child interaction

## Abstract

Prior evidence supports that the home environment is related to children’s development of school readiness skills. However, it remains unclear how construct- and timing-specific aspects of the home environment are related to children’s school readiness skills, unique from overall, stable aspects of home quality. Unpacking associations due to specific constructs and timing of the home environment may provide insights on the theoretical processes that connect the home environment to school readiness. Using data from the NICHD Study of Early Child Care and Youth Development (*N* = 1,364), the current study examines how timing (36 and 54 months) and constructs (educational *stimulation* and socio-emotional *responsivity*) of the home environment, relative to overall levels across time, relate to children’s language skills, math skills, and externalizing behaviors. The overall, stable aspects of the home environment were significantly associated with children’s language skills and externalizing problems. Additionally, there were significant paths from the stimulation construct at 54 months to math skills, language skills, and externalizing problems. These findings provide evidence that although the overall home environment is predictive of school readiness, the stimulation construct of the home environment at 54 months has additional concurrent relations to children’s school readiness. Implications for the role of the home environment and children’s school readiness are discussed.

## Introduction

Preschoolers’ language ability, math skills, and externalizing behaviors are key indicators of school readiness and are predictive of children’s success in the formal school environment (Ramey and Ramey, 2004; High, 2008). Language is one of the most important skills for learning and is foundational for reading development and later academic achievement (Chomsky, 1972; Whitehurst et al., 1988; Durham et al., 2007; Dickinson et al., 2010). Early math skills before kindergarten entry are an important predictor of later achievement in both math and reading (Duncan et al., 2007; Watts et al., 2014). Additionally, children exhibiting fewer externalizing behaviors tend to be more successful at following rules and developing positive social relationships with peers and teachers when they start school (Ladd and Sechler, 2012; Roskam, 2018), and having positive relationships with teachers is related to long-term student success (Hamre and Pianta, 2001; Burchinal et al., 2002). Although the home environment has been related to these skills and behaviors in prior research (Leventhal et al., 2004) there still remains a gap in understanding how the specific constructs and timing of the home environment relate to children’s school readiness.

Using a longitudinal dataset, the current study examined the associations between children’s early home environments and school readiness skills (i.e., math, language, externalizing behaviors). Specifically, we examine to what extent these associations vary as a function of specific constructs (i.e., educational stimulation and socio-emotional responsivity) and specific timing (i.e., 36 and 54 months) of the home environment relative to overall, stable aspects of home quality. Educational *stimulation* is a specific aspect of the home environment that refers to experiences that promote cognitive development (e.g., parent encouraging child to read and learn numbers). Socio-emotional *responsivity* is a specific aspect of the home environment that refers to experiences that support socio-emotional development (e.g., parent praising child). Addressing these issues will provide insight into how the home environment contributes to children’s school readiness. For instance, it addresses whether specific constructs of the home environment are uniquely associated with each school readiness skill beyond what is common across the constructs. Similarly, this study examines the relative associations due to the specific timing of experiences (i.e., 36 and 54 months) that go beyond what is common across time.

Theoretically, it is important to understand whether the specific constructs and timing of experiences are uniquely related to children development of school readiness beyond overall levels of home environment quality. Specifically, the estimates that are time- and construct-specific may be less biased when controlling for overall, stable levels of home quality. This is because the overall, stable levels of the home environment are subjected to potential omitted variable biases (i.e., stable characteristics of the child or family that influence both the home and child outcomes). In models that do not control for overall, stable levels, the construct- and/or time-specific estimates would be subject to these same biases. Disentangling these connections provides insight for developmental theories on how the timing of different kinds of experiences contribute to children’s school readiness skills, and potentially provide insights on the types of interventions or experiences that would be most impactful for promoting school readiness. To the authors’ knowledge, no studies to date have simultaneously examined how specific constructs of the home environment at different developmental time points are related to construct specific school readiness skills, while accounting for the overall, stable levels of the home environment.

### Overall Levels Versus Specific Constructs of the Home Environment for Language, Math, and Externalizing Behaviors

A number of studies have provided evidence that the home environment contributes to the development of various school readiness outcomes (Bradley et al., 1989; Bradley, 1993; Jackson et al., 2000; National Institute of Child Health,, 2000; Forget-Dubois et al., 2009; Hartas, 2016). Studies measuring overall levels of the home environment have found strong associations between the home environment and children’s language development (Elardo et al., 1977; Gottfried and Gottfried, 1984; Storch and Whitehurst, 2001; Connor et al., 2005; Foster et al., 2005) such that children exposed to cognitively stimulating and supportive home environments have higher language competence. High quality home environments are also predictive of young children’s math achievement (Melhuish et al., 2008; Anders et al., 2012; Young-Loveridge, 1989) and fewer externalizing behavior problems (Jackson et al., 2000; Fanti and Henrich, 2007, 2010; Price et al., 2013). These studies provide support for theoretical claims that young children’s home environment, which is composed of the quality and quantity of cognitive stimulation and emotional support in a safe physical environment (Bradley, 1993; Linver et al., 2004) contributes to school readiness skills. However, it is unknown to what extent specific constructs of the home environment are differentially related to children’s language, math, and externalizing behaviors while accounting for associations due to the overall home environment.

Correlational studies have developed and used subscales of the early home environment to find that most or all constructs of the home environment are associated with children’s intelligence and achievement scores at the start of school (Bradley, 1993). In one study, Leventhal et al. (2004) used five large-scale data sets and found that subscales measuring learning stimulation and access to reading in the home environments of 3-year-old were most robustly associated with children’s cognitive and behavioral outcomes at 5-year-old. Additionally, researchers have focused on measuring domain-specific aspects of the home environment, such as the home literacy environment or the home numeracy environment, and have found that domain-specific home environments are predictive of preschoolers’ language ability and numeracy skills (Melhuish et al., 2008; Anders et al., 2012; Niklas and Schneider, 2015; Lehrl et al., 2020). Further, specific aspects of the home environment, such as maternal negative behavior and lack of home organization, are related to externalizing problems in young children transitioning into elementary school (Eamon, 2000; Price et al., 2013; Yildirim and Roopnarine, 2015). Although these studies provide evidence that construct-specific home environments are strongly associated with specific child outcomes, these studies do not tease apart the extent to which the relations between specific constructs of the home environment and child outcomes are unique or due to shared variance of the overall quality in the home environment. Specifically, certain constructs (e.g., educational stimulation) may be more correlated with outcomes because they are also more closely related to the overall home environment, and not uniquely due to the specific construct. If that is the case, the estimates for specific constructs of the home would be confounded by the overall levels in home quality (and any omitted variables that impact overall levels in home quality and the outcome).

### Overall Levels Versus Specific Timing of the Home Environment for Language, Math, and Externalizing Behaviors

Many studies on the home environment support the longitudinal explanation that early experiences in the home are related to school readiness skills and future academic outcomes (Elardo et al., 1977; Connor et al., 2005; Mccarty et al., 2005; Melhuish et al., 2008; Fanti and Henrich, 2010). For example, research shows that the home environment at 54 months of age was predictive of language skills at 54 months of age, as well as literacy skills at the end of first grade (Connor et al., 2005). Another study suggests that the home environment at 5 years of age predicted numeracy skills concurrently and at age 7 (Melhuish et al., 2008). Further, research suggests that the early home environment measured at 6 and 15 months of age predicted externalizing problems measured between 2 and 12 years of age (Fanti and Henrich, 2010).

Previous studies have focused on longitudinal relations between the early home environment and school readiness skills that develop before formal school entry (Elardo et al., 1977; Senechal and LeFevre, 2002; Roberts et al., 2005; Torppa et al., 2007; Gonzalez et al., 2010; Napoli and Purpura, 2018; Susperreguy et al., 2020). Additionally, strong concurrent associations have been found between the home learning environment and school readiness skills (Connor et al., 2005; Rodriguez et al., 2009; Son and Morrison, 2010; Anders et al., 2012; Cristofaro and Tamis-LeMonda, 2012). It is unknown, however, whether the specific timing of experiences in the early home environment (i.e., experiences at 36 or 54 months) are uniquely related to school readiness skills beyond stability in the quality of the home environment. This is important because if associations reported are primarily due to stability in the home environment, then the associations implied are subjected to omitted variable bias that exert influences on both the stability of the home environment and children’s school readiness. Conversely, if associations emerge with school readiness skills that are unique to a specific time period in development and not overall stability in home quality, it is likely a less biased estimate of that association because omitted variables that exert stable influences on home quality and child outcomes are controlled for. Thus, we are unpacking whether associations are due to variations at specific points in children’s development unique from stability in the home environment quality; here the omitted variables that have stable influences on the home environment and school readiness skills are controlled for in the model by the overall, stable home factor (though time-specific confounds remain a concern).

## Current Study

The aim of the current study is to simultaneously examine the relations between construct- and timing-specific aspects of the home environment and children’s school readiness skills. The current study extends previous literature by examining the extent to which associations between the home environment and preschooler’s math, language, and externalizing behaviors vary as a function of specific constructs (i.e., stimulation and responsivity) and the specific timing (i.e., 36 and 54 months) of the home environment relative to overall, stable levels across time. This study addresses to what extent the stimulation and responsivity constructs at 36 and 54 months of the home environment differentially relate to children’s school readiness outcomes when holding constant overall, stable aspects of the home environment.

## Materials and Methods

### Participants

A sample of 1,364 children (52% were male) from the NICHD Study of Early Child Care and Youth Development was used for this study. Participants were recruited during hospital visits with mothers at birth of their infant in 1991 across 10 sites in the United States (Little Rock, AR; Irvine, CA; Lawrence, KS; Boston, MA; Hickory, NC; Philadelphia, PA; Pittsburgh, PA; Charlottesville, VA; Seattle, WA; Madison, WI). Majority of the mothers were European American (80%) and they averaged 14.23 years of education (i.e., the average mother completed a little more than 2 years of college). The maximum for years of education was 21 years and means that a mother completed 5 years beyond a bachelor’s degree. See Table 1 for summary statistics of demographic characteristics.

**TABLE 1:** Descriptive statistics for variables included in the study.

Home Scales	N	M	SD	Min	Max
Learn 36	1179	7.16	2.52	0	11
Language 36	1179	6.02	1.14	0	7
Academic 36	1179	3.37	1.22	0	5
Responsive 36	1179	5.61	1.36	0	7
Modeling 36	1179	3.17	1.13	0	5
Acceptance 36	1179	3.39	0.92	0	4
Learn 54	1039	9.43	1.53	1	11
Language 54	1044	6.62	0.71	1	7
Academic 54	1045	3.86	1.06	0	5
Responsive 54	1044	5.23	1.29	0	7
Modeling 54	1043	3.51	1.03	0	5
Acceptance 54	1044	3.61	0.75	0	4
**Outcomes**					
Language 54	1053	99.63	20.39	50	137
Math 54	1053	102.94	15.63	41	153
Externalizing 54	1061	51.69	9.39	30	82
**Covariates**					
Male	1364	0.52	0.50	0	1
White	1364	0.80	0.40	0	1
Black	1364	0.13	0.34	0	1
Hispanic	1364	0.05	0.21	0	1
Other	1364	0.02	0.14	0	1
Father in Home	1305	0.82	0.35	0	1
Family income	1302	3.62	2.87	0.14	22.47
Mom Vocabulary	1167	99.01	18.35	40	159
Mom Education	1363	14.23	2.51	7	21
Externalizing 24	1189	52.32	8.48	30	89
MDI 24	1162	92.15	14.64	50	150
Vocabulary 24	1073	44.27	29.43	0	99

### Measures

#### Early Childhood Home Observation for Measurement of the Environment Inventory

Children’s home environments were measured using the Early Childhood Home Observation for Measurement of the Environment Inventory (EC-HOME) at 36 and 54-month-old (Caldwell and Bradley, 1984). The EC-HOME is a reliable and valid measure for the preschool age range (Bradley, 1994). The EC-HOME is composed of 55 items clustered into eight subscales: (1) Learning Materials, (2) Language Stimulation, (3) Physical Environment, (4) Responsivity, (5) Academic Stimulation, (6) Modeling, (7) Variety, and (8) Acceptance. However, this study focuses on the six subscales (Learning Materials, Language Stimulation, Responsivity, Academic Stimulation, Modeling, and Acceptance) that are theoretically important for cognitive and behavior outcomes. These six subscales are separated into the stimulation and responsivity constructs. The *stimulation* construct consists of Learning Materials (e.g., child has educational toys, games, books), Language Stimulation (e.g., parent encourages verbal communication and vocabulary development), and Academic Stimulation (e.g., child is encouraged to read, learn colors, learn numbers, etc.) which are subscales that represent the quality of cognitive stimulation available to the child at home. The *responsivity* construct consists of Responsivity (e.g., parent hugs child, answers child’s questions, praises child), Modeling (e.g., parent allows child to express negative emotions without retaliation), and Acceptance (e.g., parent does not spank child), which are subscales that represent the quality of social/emotional support and responsivity available at home. The EC-HOME was collected during home visits using direct observation and semi-structured interviews with mothers. All observers maintained >90% agreement with the master coder at both time points. The alpha coefficient for the total EC-HOME score is 0.93 with alphas for subscales ranging from 0.53 to 0.88.

#### Preschool Language Scale—3

Children’s language outcomes were directly assessed using the Preschool Language Scale-3 (PLS-3; Zimmerman et al., 1979). The PLS-3 assessed vocabulary, grammar, morphology, and language reasoning at 54 months of age. The test is comprised of two parts: (a) the auditory comprehension scale that measures what children “know” or understand, but may not “say,” and; (b) the expressive communication scale that assesses what children actually say or produce. Items are scored as 1 for each question if the pass criterion is met or if the child self-corrects a response. A score of 0 is given for each item if the pass criterion is not met or for partially correct or incomplete responses. Raw scores are computed for each subscale by subtracting the number of “0” scores after the “true” basal from the number of the last subscale task administered (i.e., the “true” ceiling). The PLS-3 standard scores have a normed mean of 100 and a standard deviation of 15 and were used in this study.

#### Woodcock-Johnson Applied Problems

Children’s math outcomes were directly assessed at 54 months using the Woodcock-Johnson Applied Problems (Woodcock and Johnson, 1989). This instrument is valid and reliable for this age range (McGrew et al., 1991). Applied Problems assesses children’s ability to solve mathematical problems that include basic counting, addition, subtraction, and multiplication primarily through word problems read to the child. In order to solve the problems, the subject must recognize the procedure to be followed and then perform relatively simple calculations. Each assessment item is scored as 1 (correct response) or 0 (incorrect or no response) and the raw score is the total number of correct responses. Standard scores, which are based on a normed mean of 100 and a standard deviation of 15, were used in analyses.

#### Child Behavior Checklist/4-18

Children’s externalizing behavior was measured using the parent/caregiver reported Child Behavior Checklist/4-18 (CBCL/4-18) at 54 months of age (Achenbach and Edelbrock, 1991). The CBCL/4-18 is the most widely used screening instrument available for tracking the emergence of behavior problems in children. The CBCL includes items that illustrate childhood behavioral and emotional problems which were selected from previous literature, as well as interviews with parents and mental health professionals. Mothers were asked to rate 33 externalizing items about how characteristic each behavior was of their child over the last 2 months (0 = not true, 1 = sometimes true, 2 = very true).

#### Covariates

Control variables include measures of children’s mental development, language skills, and externalizing behavior when they were 24 months old. Measures of mothers’ vocabulary knowledge when their child was 36 months old was also used as a control variable. Additionally, race, gender, family composition (e.g., father lives with mother), and family income were included as controls.

#### Bayley Scales of Infant Development—Revised

Children’s level of cognitive development was directly assessed using the Revised Bayley Scales (Bayley, 1991). The Mental Development Index (MDI) of the Bayley was used to assess cognitive skills (e.g., memory, early verbal communication, problem solving, etc.) at 24 months of age. The MDI is one of the most widely used and valid measure of cognitive ability.

#### MacArthur Communicative Development Inventories (CDI) for Infants and Toddlers

Mothers’ reported on their child’s vocabulary production at 24 months of age using the CDI/Toddler (Fenson et al., 1991). The CDI checklist measured words that children used, as well as, syntactic/morphological development and nominal/pronominal style. Internal consistency for this measure was 0.96. This measure includes two parts. The first consists of a 680 word vocabulary production checklist, organized into 22 semantic categories such as “animals” and action word followed by five questions aimed at assessing the child’s ability to differentiate past, future, and absent objects and events. The second part consists of 125 items that are designed to assess syntactic and morphological development, as well as nominal/pronominal style.

#### Child Behavior Checklist/2-3

Children’s externalizing behavior was measured using the parent/caregiver reported Child Behavior Checklist/2-3 (CBCL/2-3) at 24 months of age (Achenbach and Edelbrock, 1991). Mothers were asked to rate 99 items describing child’s behavioral problems over the last 2 months (0 = not true, 1 = sometimes true, 2 = very true).

#### Peabody Picture Vocabulary Test—Revised

Mothers’ language outcomes were directly assessed using the Peabody Picture Vocabulary Test – Revised (PPVT-R; Dunn and Dunn, 1981). The PPVT-R assessed mothers’ receptive vocabulary knowledge when their child was 36 months of age. Participants selected one of four pictures that represented each vocabulary word they were presented. Internal consistency for this measure ranged from 0.80 to 0.83. The PPVT-R consists of 175 plates with four pictures on each plate. Plates are arranged in increasing order of difficulty. PPVT-R standard scores were used for analyses.

### Analytic Strategy

All data management and descriptive analyses were run in Stata 15 (StataCorp,, 2017) and all structural equation models were run in Mplus 8 (Muthén and Muthén, 2017). Our analyses begin by descriptively examining the aspects of the HOME scale at 36 and 54 months. Before running our structural equation model with path estimates to address our primary research question, we ran a series of factor models to determine which conceptual model best fit the HOME data. Specifically, we test whether the data support (1) a random intercept HOME factor, (2) a random intercept HOME factor with time-specific factors (36 versus 54 months), (3) a random intercept HOME factor with construct-specific factors (stimulation versus responsivity), or (4) a random intercept HOME factor with time- and construct-specific factors. The random intercept HOME factor with time- and construct-specific factors would allow us to investigate the associations between time- and construct-specific factors and school readiness, while controlling for the overall, stable HOME factor (the random intercept).

Once the final factor model was selected for the HOME, a comprehensive set of covariates are included as predictors of each of three school readiness outcomes (i.e., math, language, and externalizing) along with the HOME factor/s. Time- and construct-specific factors are included one at a time to determine if they relate to the outcomes above and beyond the overall, stable HOME random intercept factor and covariates. As a robustness check to the final model, covariates for children’s cognitive, language, and externalizing at 24 months were removed. This was done to test whether our pattern of results is changed when examining overall levels in externalizing, language, and math versus when controlling for prior knowledge and behavior at 24 months.

All analyses used full information maximum likelihood (FIML) to address issues related to missing data (see Table 1 for the number of observations for all variables included in analyses). Although relatively little missing data occurred on any of the variables, FIML is a recommended strategy that uses all available information to provide less biased estimates than restricting to only cases that provide complete data (Acock, 2012).

## Results

### Descriptive Statistics and Correlations

The number of observations, means, standard deviations, and ranges for all variables are included in Table 1. In general, HOME scales tended to be positively endorsed with higher scores at 54 months compared to 36 months. Language, math, and externalizing scores were close to nationally normed averages (100 for language and math; 50 for externalizing), suggesting the sample is representative of typical development during this age period. Correlations between the HOME scales and children’s outcomes are included in Table 2. All HOME scales were significantly correlated with one another and significantly correlated with each of the three school readiness skills. Additionally, families with higher incomes and mothers with more education and better vocabulary tended to have higher HOME scores and children with better school readiness skills.

**TABLE 2:** Correlations for variables included in the study.

	1	2	3	4	5	6	7	8	9	10	11	12	13	14	15
(1) Learn 36															
(2) Language 36	0.51*														
(3) Academic 36	0.53*	0.53*													
(4) Responsivity 36	0.36*	0.37*	0.33*												
(5) Modeling 36	0.38*	0.32*	0.26*	0.29*											
(6) Acceptance 36	0.29*	0.21*	0.15*	0.23*	0.33*										
(7) Learn 54	0.61*	0.33*	0.34*	0.29*	0.29*	0.25*									
(8) Language 54	0.32*	0.39*	0.30*	0.21*	0.19*	0.16*	0.34*								
(9) Academic 54	0.36*	0.37*	0.39*	0.18*	0.19*	0.12*	0.37*	0.44*							
(10) Responsivity 54	0.26*	0.19*	0.17*	0.31*	0.17*	0.16*	0.29*	0.29*	0.18*						
(11) Modeling 54	0.31*	0.26*	0.22*	0.22*	0.30*	0.25*	0.32*	0.29*	0.23*	0.30*					
(12) Acceptance 54	0.19*	0.14*	0.08*	0.12*	0.20*	0.31*	0.23*	0.18*	0.15*	0.18*	0.29*				
(13) Language 54	0.47*	0.31*	0.30*	0.32*	0.27*	0.23*	0.45*	0.28*	0.32*	0.29*	0.27*	0.24*			
(14) Math 54	0.42*	0.24*	0.24*	0.27*	0.20*	0.23*	0.36*	0.21*	0.24*	0.23*	0.19*	0.21*	0.70*		
(15) Externalizing 54	−0.14*	−0.10*	−0.09*	−0.10*	−0.14*	−0.17*	−0.21*	−0.08*	−0.10*	−0.11*	−0.17*	−0.20*	−0.14*	−0.06*	
Male	−0.09*	–0.05	−0.06*	–0.04	−0.06*	−0.07*	–0.05	–0.05	−0.06*	−0.09*	–0.01	−0.08*	−0.15*	−0.12*	−0.08*
White	0.37*	0.17*	0.17*	0.21*	0.19*	0.08*	0.28*	0.10*	0.08*	0.24*	0.20*	0.10*	0.33*	0.28*	–0.05
Black	−0.36*	−0.11*	−0.17*	−0.18*	−0.16*	−0.09*	−0.31*	−0.10*	−0.09*	−0.24*	−0.20*	−0.12*	−0.34*	−0.32*	0.05
Hispanic	−0.11*	−0.10*	–0.03	−0.09*	−0.08*	–0.02	–0.04	–0.01	0.01	–0.03	–0.03	0.00	–0.05	–0.02	0.02
Other	–0.03	−0.08*	–0.03	–0.03	–0.03	0.02	–0.02	–0.02	–0.03	–0.05	–0.05	0.02	–0.04	0.00	–0.00
Father in Home	0.38*	0.18*	0.18*	0.26*	0.21*	0.16*	0.34*	0.13*	0.10*	0.19*	0.19*	0.13*	0.25*	0.25*	−0.10*
Family income	0.39*	0.20*	0.17*	0.24*	0.26*	0.17*	0.39*	0.16*	0.16*	0.20*	0.21*	0.16*	0.39*	0.33*	−0.10*
Mom Vocabulary	0.47*	0.26*	0.23*	0.26*	0.30*	0.21*	0.42*	0.18*	0.16*	0.28*	0.26*	0.23*	0.49*	0.44*	−0.12*
Mom Education	0.48*	0.26*	0.25*	0.30*	0.34*	0.24*	0.48*	0.19*	0.22*	0.27*	0.27*	0.26*	0.46*	0.39*	−0.14*
Externalizing 24	−0.22*	−0.11*	–0.12	−0.14*	−0.16*	−0.17*	−0.24*	−0.09*	−0.14*	−0.17*	−0.15*	−0.18*	−0.26*	−0.22*	0.55*
MDI 24	0.42*	0.24*	0.26*	0.28*	0.28*	0.20*	0.34*	0.25*	0.26*	0.26*	0.24*	0.19*	0.64*	0.57*	−0.07*
Vocabulary 24	0.25*	0.17*	0.20*	0.14*	0.11*	0.02	0.18*	0.20*	0.21*	0.05	0.09*	0.03	0.35*	0.28*	–0.03

### Factor Structure of the HOME at 36 and 54 Months

The factor structure of the HOME was tested in multiple ways before selecting the final measurement model used in the structural equation models that included outcomes and control variables that addressed the primary research question of interest. First, all indicator variables (scales) were standardized so the overall factor (random intercept) could represent what is equally shared across all scales independent of scaling characteristics (scales with more or fewer items included) and across both time points. Second, residual correlations were included between all common scales assessed at each time point (e.g., 36-month academic stimulation with 54-month academic stimulation). Next, a series of models were run that examined comparison of model fit between models that specified (1) a HOME random intercept only, (2) a HOME random intercept with time-specific factors (all 36 months scales loading onto a common factor), (3) a HOME random intercept with construct-specific factors (e.g., academic stimulation, language stimulation, and learning materials all loading onto a common factor, called stimulation), and (4) a HOME random intercept model with time- and construct-specific factors.

Fit indices for the measurement models are presented in Table 3. Fit indices improved with each specification, ultimately supporting the model with time- and construct-specific factors. However, this model (Model 4 in Table 3) included all non-significant loadings onto the responsivity factor at 54 months. Removing this factor (model 5) resulted in improved fit in terms of the Bayesian Information Criteria (BIC) and was selected as the measurement model for the primary analyses (Kline, 2005). Correlations between the factors for stimulation and responsivity at 36 months and between the factors for 36- and 54-month stimulation were included. A correlation between responsivity at 36 months and stimulation at 54 months was tested but found to be non-significant and was therefore excluded in the primary structural equation models.

**TABLE 3:** Fit indices for measurement models of the home scales.

Measurement Models		(df) x^2^	p	CFI	RMSEA	BIC
(1)	Random Intercept Only	(59) 632.77	0.000	0.824	0.090	35354
(2)	RI + Time-Specific Factors	(46) 172.93	0.000	0.961	0.048	34987
(3)	RI + Construct-Specific Factors	(46) 232.66	0.000	0.943	0.058	35046
(4)	RI + Time- and Construct-Specific Factors	(43) 129.23	0.000	0.974	0.041	34964
(5)	Model 4 without the 54 months Responsivity Factor	(48) 146.25	0.000	0.970	0.041	34946

### Time- and Construct-Specific Associations Between HOME and Children’s School Readiness

**FIGURE 1:**
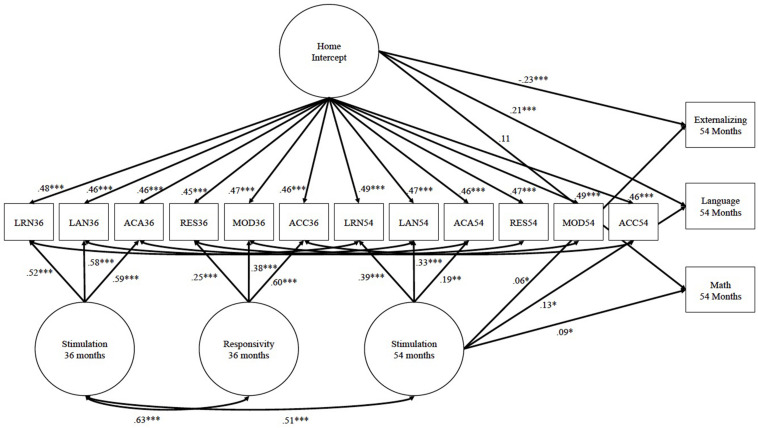
Associations between home factors and children’s math, language, and externalizing. All estimates are standardized. Control variables include: gender, race, whether the father was in the home, family income, mother vocabulary, mother education, externalizing behaviors at 24 months, mental development index at 24 months, and vocabulary skills at 24 months. ^∗^*p* < 0.05, ^∗∗^*p* < 0.01, and ^∗∗∗^*p* < 0.001.

Once the final measurement model for the HOME scales was selected, covariates were entered along with the HOME factor/s as predictors of children’s math, language, and externalizing behaviors at 54 months. Controls for cognitive, language, and externalizing at 24 months were included to control for earlier skills and behaviors that likely contribute to these school readiness skills at 54 months. The overall HOME factor was included in all models to control for the influence of shared variance across HOME scales, across time. The time- and construct-specific factors were entered one at a time on each outcome with all significant associations included in the final model (see Figure 1). The final model explained 43, 31, and 35% of the variance for language, math, and externalizing behaviors, respectively.

The overall HOME factor was significantly associated with 54 months language (β = 0.21, *p* < 0.001), and externalizing (β = −0.23, *p* < 0.001), such that overall better home environments (regardless of construct or time point) were associated with increases in language abilities and decreases in externalizing behaviors. The stimulation factor at 54 months had additional significant associations with math (β = 0.09, *p* = 0.047), language (β = 0.13, *p* = 0.023), and externalizing (β = 0.06, *p* = 0.049). This suggests that beyond the degree to which the overall home factor influences learning materials, academic stimulation, and language stimulation at 54 months, this construct- and time-specific factor also relates to children’s school readiness development uniquely. It should be noted that the association for stimulation at 54 months to externalizing is in the opposite direction than the overall HOME factor, suggesting that the association between HOME scales other than stimulation at 54 months (e.g., scales from the responsivity construct) are more directly related to reductions in externalizing than the HOME scales contributing to stimulation at 54 months. Conceptually this makes sense, as the overall HOME factor consists of scales that represent the responsivity construct (i.e., responsivity, modeling, and acceptance) at both 36 and 54 months which are hypothesized to be more related to reductions in externalizing, though it also includes early stimulation scales at 36 months (which may be related to reductions in externalizing behaviors as well). See Table 4 for all unstandardized estimates from the model.

**TABLE 4:** Unstandardized estimates from model.

	Math			Language			Externalizing		
	*B*	*SE*	*p*	*B*	*SE*	*p*	*B*	*SE*	*p*
HOME RI	3.24	1.76	0.065	7.94	1.17	0.000	−4.47	1.09	0.000
Stimulation 54	5.39	2.25	0.017	9.92	4.56	0.030	2.19	1.25	0.079
Male	−1.11	0.61	0.067	−2.88	0.88	0.001	−2.04	0.40	0.000
Black	−3.60	1.17	0.002	−4.28	1.87	0.022	−1.36	0.91	0.134
Hispanic	0.76	2.18	0.737	−1.36	2.04	0.507	−0.79	1.32	0.547
Other	1.68	2.34	0.473	−4.36	3.98	0.273	0.02	2.12	0.991
Father in Home	0.57	1.25	0.650	−1.09	0.84	0.194	0.08	1.03	0.941
Family income	0.17	0.13	0.184	0.42	0.17	0.011	0.06	0.09	0.522
Mom Vocabulary	0.15	0.03	0.000	0.21	0.05	0.000	−0.00	0.02	0.881
Mom Education	0.33	0.16	0.039	0.42	0.23	0.073	−0.04	0.15	0.811
Externalizing 24	−0.08	0.04	0.018	−0.12	0.05	0.017	0.61	0.05	0.000
MDI 24	0.42	0.07	0.000	0.54	0.06	0.000	0.06	0.03	0.026
Vocabulary 24	0.01	0.02	0.611	0.05	0.02	0.015	0.00	0.01	0.901

*HOME RI = random intercept HOME factor.*

#### Removing 24 Months Cognitive, Language, and Externalizing Controls

We conducted a sensitivity check by removing the control variables for cognitive, language, and externalizing behaviors at 24 months to see if model conclusions changed. The only substantive change that occurred is that the stimulation factor at 36 months was significantly associated with language (β = 0.11, *p* = 0.028) and math outcomes (β = 0.12, *p* = 0.041) above and beyond the HOME random intercept. However, stimulation at 54 months was also significantly associated with language (β = 0.20, *p* = 0.003) and math outcomes (β = 0.14, *p* = 0.011) above and beyond the HOME random intercept, and once the 36 and 54 stimulation factors were included simultaneously, only the 54-month stimulation factor paths remained significant for math and language. Thus, model conclusions (with regard to significant associations) were very similar regardless of whether 24-month-old skills and externalizing behavior variables were included.

## Discussion

This study examined the relation between construct- and time-specific aspects of the home environment and preschooler’s development of school readiness skills. The results suggest that overall, stable home environment quality was positively associated with language skills and negatively associated with externalizing behaviors. Independent of the overall, stable HOME factor, results also indicate that the stimulation construct of the home environment at 54 months of age was significantly related to language skills, math skills, and externalizing behaviors. These findings are potentially less biased because they are independent of factors (i.e., omitted variable biases) that exert stable influences on both home environments and school readiness skills. They provide evidence that the stimulation construct of the home environment at 54 months has unique relations to children’s math and language development, as well as externalizing behaviors (though these were in the opposite direction than the overall home factor). Prior work on the theoretical connections between specific aspects of the home environment and children’s school readiness has yielded challenging to interpret associations due to the shared variance across constructs in home quality (Melhuish et al., 2008; Anders et al., 2012; Lehrl et al., 2020). This has been similarly, problematic when measuring the home environment across time points but not fully accounting for what is relatively stable across time. The current study addresses these issues, yielding support for strong associations between the overall home environment and externalizing behaviors and language at 54 months, with construct- and time-specific associations between 54-month stimulation and children’s language and math abilities.

### Construct-Specific and Timing-Specific Associations Between the Home Environment and School Readiness

Results of the current study suggest that there is a construct and time-specific association between the stimulation construct and children’s language and math skills. The current study found that the stimulation construct at 54 months was uniquely associated with children’s language and math skills, above and beyond the quality of the overall home environment (i.e., 36 to 54 months). It is not surprising that the quantity and quality of learning materials, language stimulation, and academic stimulation at 54 months are likely important for children’s skills at 54 months. This finding is consistent with previous studies providing support for the relation between construct-specific home learning activities and children’s language and math skills (Manolitsis et al., 2013; Skwarchuk et al., 2014) although these studies did not account for shared variance in home experiences across constructs. For example, home literacy activities are predictive of language development and home numeracy activities are predictive of math development (Senechal and LeFevre, 2002; Skwarchuk et al., 2014). Additionally, our results support a concurrent association which is likely stronger because home stimulation and school readiness skills were measured during the same period of time. It is important to note that the size of effects of the relation between home activities and child skills in the present study appear to be smaller than the size of effects in previous studies, though the current study may provide less biased estimates because it controls for overall levels of the home environment across time.

Previous research also suggests that parents may engage their preschool aged children in more advanced learning activities as they get closer to school entry (Thompson et al., 2017). It is possible that specific parent-child interactions that take place when children are 54 months are more proximal to academic school readiness skills. The idea that specific parent-child interactions at 54 months are more proximal to academic school readiness skills seems to particularly be the case for the relation between the home environment and children’s math skills. Although the stimulation construct at 54 months was related to math skills, the overall home environment across time was not significantly related to math skills. It may be that parents begin engaging children in more math related activities when they are closer to approaching school entry (e.g., more advanced math activities when child is 4 years old rather than 3 years old; Thompson et al., 2017). Additionally, cross-domain associations supporting positive relations between numeracy activities and children’s language outcomes, as well as positive relations between literacy activities and children’s math outcomes have been found likely due to the shared variance across domains and the role of language and how it underlies the development of early math skills (LeFevre et al., 2010; Anders et al., 2012; Napoli and Purpura, 2018).

In contrast, both the overall home environment across time points and the stimulation construct at 54 months (above and beyond the overall factor) are related to children’s language skills. Although language stimulation is important well before preschool age (Hirsh-Pasek et al., 2015) parents may engage children in more advanced language and literacy activities as their child’s language acquisition grows and the child approaches school entry, which is consistent with scaffolding theories (Vygotsky, 1978). This study along with previous studies further supports the theoretical importance of the quantity and quality of learning materials, language stimulation, and academic stimulation being present in the home environment specifically to support the development of children’s skills during the preschool period.

Somewhat unexpectedly, the stimulation construct at 54 months was positively associated with externalizing behaviors, however, the overall home environment was negatively associated with externalizing behaviors (and substantially larger in magnitude). These two results need to be interpreted simultaneously, such that aspects of the home environment other than 54-month stimulation (i.e., 36-month stimulation and responsivity, and 54-month responsivity) were more closely associated with reductions in externalizing behaviors than was 54-month stimulation. Notably, bivariate correlations indicate that 54-month stimulation variables were negatively correlated with externalizing behaviors (Table 2), thus the direction of associations changes only when simultaneously considering the overall, stable HOME factor as well. In this regard, these findings are not surprising and are consistent with hypotheses. This is also consistent with prior research that a better overall home environment is related to decreased behavior problems (Jackson et al., 2000; Fanti and Henrich, 2010).

## Limitations and Future Directions

Although the results of the current study yield important insights regarding the construct- and timing-specific relations between the home environment and preschoolers school readiness outcomes, limitations of the study and areas for future research should be noted. One limitation is that this study uses a non-experimental design, and therefore, causal implications cannot be inferred. Additionally, there may be child effects and omitted variable bias that accounts for the obtained findings. For example, children with more advanced language, math, and social skills may elicit more responsive and stimulating engagement from parents in their home environment. However, a key advantage of this the current study is that it advances on previous studies that have not controlled for the overall levels and stability in home environments when making time- and construct-specific assertions. Although we controlled for prior cognitive and language abilities and externalizing behaviors at 24 months, auto-regressors were not available for all school readiness skills. Additional research is needed to evaluate whether or not there are bidirectional relations between construct- and timing-specific aspects of the home environment and school readiness skills.

Additionally, our findings for 54-month stimulation just reached traditional thresholds for statistical significance in most instances (i.e., 0.05). The magnitudes of effect sizes were also relatively small, around 0.10. However, we think these are potentially less biased estimates and should be considered within the overall rigor of the analytic models that teased apart shared variance across constructs and time of the home measures. Regardless, we encourage future evaluation and replication to understand if these associations remain in other samples and when using other instruments that capture the quality of the home environment.

It is possible that social desirability could have influenced the way mothers interacted with their children and answered certain questions while researchers administered the home environment measure (EC-HOME) during home visits. Further, the EC-HOME did not capture the quantity and quality of other experiences that children have in their daily lives, such as interactions with fathers or siblings and experiences within early childhood education institutions, which are also related to children’s cognitive skills and behaviors (Dunn and Plomin, 1990; Shonkoff and Phillips, 2000; Bradley et al., 2001). Although many young children are enrolled in some form preschool before they begin kindergarten, this study focused on experiences within the home environment because preschoolers tend to spend more time at home and may be receiving more individualized engagement than what is possible in a typical classroom. However, the preschool environment is an important factor for school readiness and should be considered in future studies that focus on how a child’s environment is related to school readiness. Better indicators of domain-specific aspects of the home environment (e.g., home math environment) may improve this area of research. Future research should continue to investigate different aspects of the environment (e.g., home and childcare experiences) that are related to child development by using models that simultaneously control for each proximal environmental factor because children do not experience different aspects of their environment in isolation.

## Conclusion

This study contributes to the home environment literature by providing evidence for unique concurrent associations between the stimulation construct and children’s development of language and math skills. Specifically, engagement in high quality stimulating activities was related to higher language and math skills when children were 54 months old. These findings were particularly robust considering that the models controlled for overall, stable aspects of the home quality, children’s cognitive, language, and externalizing behaviors at 24 months, as well as key sociodemographic factors (i.e., mother’s education, mother’s vocabulary ability). These findings indicate that there may be specific stimulation-related activities occurring at 54 months of age that are particularly important for the development of language and math abilities, while the overall home environment (particularly the aspects other than 54-month stimulation) is important for the reduction of externalizing behaviors. Findings suggest that researchers should be sensitive to the target construct and timing of intervention efforts for the development of some school readiness outcomes, while other outcomes may be influenced by more comprehensive interventions.

## Data Availability Statement

Publicly available datasets were analyzed in this study. This data can be found here: https://www.icpsr.umich.edu/icpsrweb/ICPSR/series/00233.

## Ethics Statement

The studies involving human participants were reviewed and approved by Internal Review Board (IRB) at Purdue University. Written informed consent to participate in this study was provided by the participants’ legal guardian/next of kin.

## Author Contributions

YK and RD contributed conception and design of the study. RD organized the database and performed the statistical analysis. All authors contributed to the writing of the manuscript, revision, read, and approved the submitted version.

## Conflict of Interest

The authors declare that the research was conducted in the absence of any commercial or financial relationships that could be construed as a potential conflict of interest.
